# Early onset and increasing disparities in neurodevelopmental delays from birth to age 6 in children from low socioeconomic backgrounds

**DOI:** 10.1186/s11689-024-09577-2

**Published:** 2024-11-05

**Authors:** Tae Hwan Han, Kyu Young Chae, Boeun Han, Ju Hee Kim, Eun Kyo Ha, Seonkyeong Rhie, Man Yong Han

**Affiliations:** 1 Department of Pediatrics, Yongin Severance Hospital, Yonsei University Health System, Yongin, Korea; 2 Department of Pediatrics, CHA Bundang Medical Center, CHA University School of Medicine, Seongnam, Korea; 3 Department of Pediatrics, Kyung Hee University School of Medicine, Seoul, Korea; 4 Department of Pediatrics, Hallym University Kangnam Sacred Heart Hospital, Seoul, Korea; 5 Department of Pediatrics, CHA University School of Medicine, 59, Yatap-ro, Bundang-gu, Seongnam-si, Gyeonggi-do 13496 Republic of Korea

**Keywords:** Neurodevelopmental delay, Socioeconomic status, Birthweight, Environmental factors, Cognition

## Abstract

**Objective:**

To analyze the complex relationship between socioeconomic status (SES) and neurodevelopmental achievements by investigating the temporal dynamics of these associations from birth to age 6.

**Methods:**

This retrospective cohort study was conducted over 6 years using population-based data from the National Health Insurance Service and integrated data from the National Health Screening Program for Infants and Children. Participants were children born between 2009 and 2011 in Korea without neurodevelopmental delays with potential developmental implications. We analyzed results from the Korean Developmental Screening Test, administered at age 6, which covered overall assessment and six domains of gross and fine motor function, cognition, language, sociality, and self-care. The secondary outcome was to determine when neurodevelopmental outcomes began after birth and how these differences changed over time.

**Results:**

Of 276,167 individuals (49.2% males), 66,325, 138,980, and 60,862 had low, intermediate, and high SES, respectively. Neurodevelopmental delays observed across all developmental domains were more prevalent in the low-SES group than in the high-SES group. Disparities in neurodevelopment according to these statuses were apparent as early as age 2 and tended to increase over time (interaction, *P* < 0.001). The cognition and language domains exhibited the most substantial disparities between SES levels. These disparities persisted in subgroup analyses of sex, birthweight, head circumference, birth data, and breastfeeding variables.

**Conclusions:**

Low SES was significantly associated with an increased risk of adverse neurodevelopmental outcomes in preschool children, particularly those affecting cognitive and language domains. These differences manifested in early childhood and widened over time.

**Supplementary Information:**

The online version contains supplementary material available at 10.1186/s11689-024-09577-2.

## Introduction

Neurodevelopment is profoundly influenced by the environments experienced by individuals, revealing disparities in resource availability [1]. Various factors including housing conditions, educational pathways, and economic well-being form social status [2]. Socioeconomic status (SES), a key measure of social standing based on income, education, or occupation, is consistently associated with neurodevelopment from the prenatal period to adulthood. Prior investigations have highlighted that lower SES can cause developmental delays, attributing them to unequal access to essential services, goods, parental support, and social interactions. Families with higher SES tend to have these resources, whereas families with lower SES encounter face obstacles, increasing the likelihood of developmental challenges [3]. 

Despite the significance of this issue, a comprehensive examination of neurodevelopmental trends across the entire population has been hindered by restricted access to medical records and systemic constraints [4]. Consequently, studies have focused on smaller cohorts within childcare facilities [5], local communities [6, 7], or specific populations such as premature infants [8–10]. Furthermore, assessments of neurodevelopment in preschool-age children have been limited, with many relying on later academic achievement as a proxy for developmental outcomes [11–13]. This gap in research has created a notable absence of studies encompassing extensive sample sizes representative of typical preschoolers.

Constraints in existing research may result from the difficulty of controlling various variables that affect neurodevelopment. These variables encompass sex [14], birthweight (BW) [15], head circumference (HC) at 4–6 months old [16], and breastfeeding practices [17], all of which have been posited to be influential determinants of neurodevelopmental trajectories. In addition, empirical evidence corroborates the significance of developmental screening assessments, which are adopted worldwide. Despite individual investigations on these aspects, there is a lack of a cohesive and comprehensive body of knowledge, even with the alignment of screening recommendations during analogous developmental stages.

Therefore, we investigated the intricate interplay between SES and neurodevelopment in preschool-aged children by assessing various aspects of neurodevelopment, including gross and fine motor functions, cognition, language, sociality, and self-care. Additionally, we aimed to identify specific points in neurodevelopment where notable differences may emerge and determine when intervention is most crucial if significant observations are noted. This knowledge is pivotal for shaping future research initiatives, policy recommendations, and interventions to bridge neurodevelopmental gaps among children.

## Materials and methods

### Study design and data sources

This investigation analyzed healthcare resource utilization data from the National Health Insurance System (NHIS) and pertinent information from the National Health Screening Program for Infants and Children (NHSPIC).

NHIS data encompass demographic attributes, such as date of birth, sex, insurance particulars, premium disbursements, residential location, and diagnostic codes (in accordance with the International Classification of Diseases, Tenth Revision codes).

The NHSPIC dataset, comprising a cohort of South Korean children (*n* = 1,420,941) born between 2009 and 2011, underwent a meticulous 6-year longitudinal examination. The NHSPIC protocol encompasses elementary inquiries and assessments, including investigations of breastfeeding practices, routine physical examinations, and evaluation of developmental milestones.

The study protocol was reviewed and approved by the Institutional Review Board of the Korea National Institute for Bioethics Policy (P01-201603-21-005). The requirement for informed consent was waived owing to the retrospective nature of this study.

### Population

**Fig. 1:**
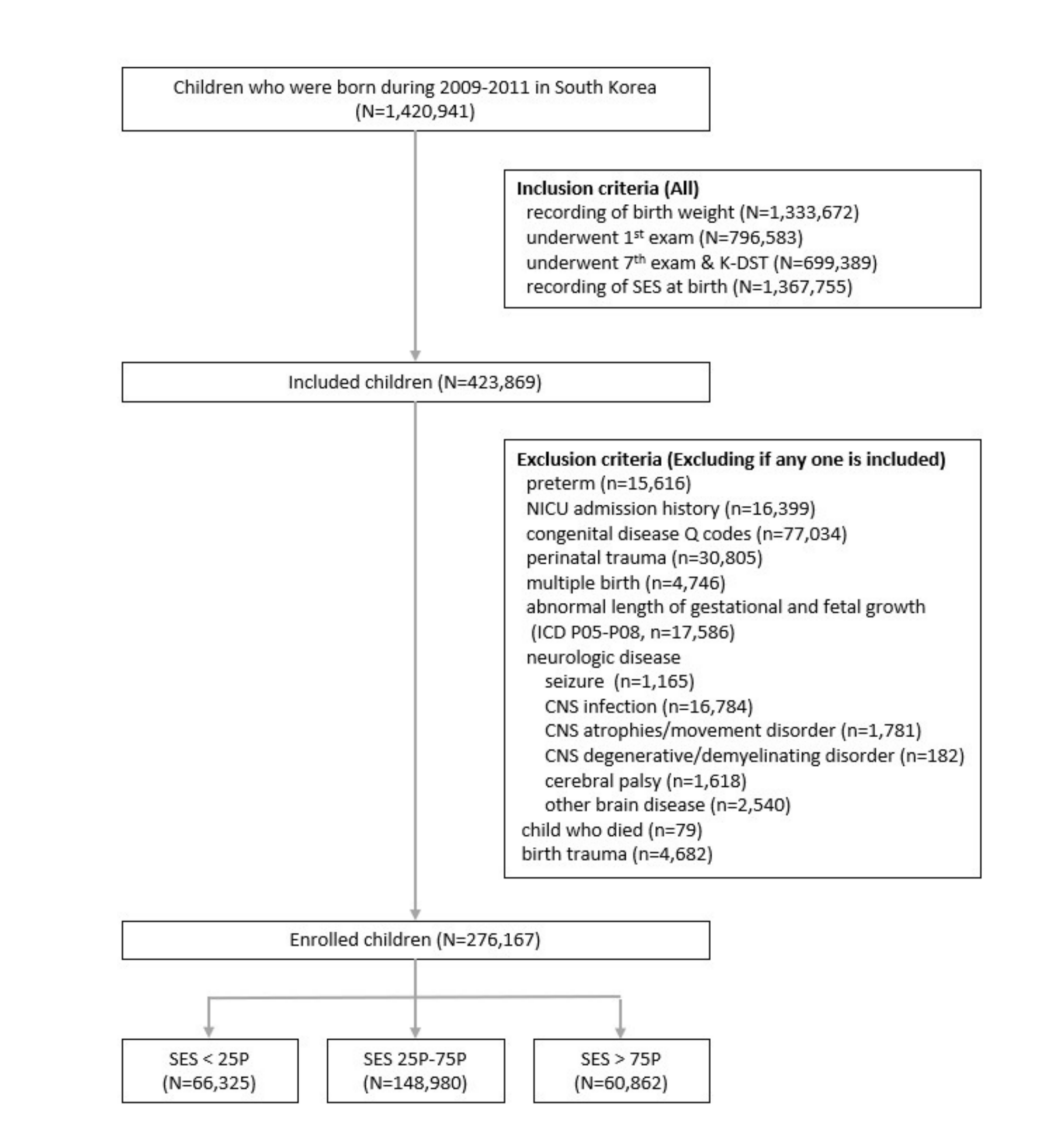
Flow diagram of study population. Socioeconomic status (SES) is categorized into three tiers based on health insurance premiums from the National Health Insurance System data. K-DST, Korean Developmental Screening Test; SES, socioeconomic status; NICU, neonatal intensive care unit; CNS, central nervous system

Between 2009 and 2011, 1,420,941 children were born in South Korea. Figure 1 illustrates the stringent inclusion criteria applied to establish a refined cohort for analysis. The inclusion criteria were as follows: recorded BW (*n* = 1,333,672), primary physical examination at 4–6 months of age (*n* = 796,583), completion of a 7th anthropometric assessment coupled with a neurodevelopmental examination utilizing the Korean Infant and Toddler Ages and Stages Questionnaire (K-ASQ) or the Korean Developmental Screening Test (K-DST) (*n* = 699,389), and possession of pertinent health insurance premium information for the computation of SES quartiles (*n* = 1,367,755).

The study cohort comprised 423,836 children. Rigorous measures were undertaken to ensure the robustness of the sample, including the exclusion of participants presenting with neurodevelopmental delays with potential developmental implications. The exclusion criteria were as follows: prematurity (*n* = 15,616), neonatal intensive care unit admission history (*n* = 16,399), diagnosed with congenital anomalies (*n* = 77,034), perinatal trauma (*n* = 30,805), multiple births (*n* = 4,746), small or large gestational age (*n* = 79), neurologic disease (*n* = 24,070), deceased (*n* = 79), and birth trauma (*n* = 4,682) (Fig. 1).

### NHSPIC

During the subject screening phase, the NHIS implemented a comprehensive series of seven iterations of the NHSPIC, targeting individuals aged 4–71 months, with specific intervals for assessments (1st, 4–6 months; 2nd, 9–12 months; 3rd, 18–24 months; 4th, 30–36 months; 5th, 42–48 months; 6th, 54–60 months; and 7th, 66–71 months). To measure neurodevelopmental function, the NHSPIC program used the K-ASQ as a developmental screening tool from 2008 to 2013 [17–19], and, in 2014, the K-DST was introduced as the primary developmental screening tool (eFigure 1). Considering the varying types of check-ups from the 2nd to the 7th iterations for neurodevelopmental measurement and the differing numbers of participants in each iteration, the assessment was conducted as an overall evaluation of the follow-up studies using both K-ASQ and K-DST for secondary outcomes, whereas the main outcome used the 7th K-DST.

### Exposure

SES was systematically categorized into three stratified tiers for subsequent data analysis: the lowest 25th percentile was defined as low SES, the 25th to 75th percentile as intermediate, and > 75th percentile as high.

The Korean health insurance system is based on the principles of a universal health insurance model, necessitating individuals or households to contribute monthly premiums proportionate to their respective income and wealth. Consequently, the magnitude of health insurance premiums serves as an indicator of a household’s financial standing. To investigate this relationship, we isolated the premium payment component from the NHIS data.

### Primary outcomes

We focused on developmental screening data derived from the 7th check-up (66–71 months of age) using the K-DST. The K-DST encompasses six domains: gross motor skills, fine motor skills, cognition, language, sociality, and self-care. Employing a comprehensive four-tiered interpretation system, the K-DST assesses developmental outcomes, categorizing them as indicative of advanced development, age-appropriate, necessitating follow-up, or warranting further evaluation [17, 18]. Within this framework, the recommendation for further evaluation is reserved for scores that fall below − 2 standard deviations.

### Secondary outcomes

The secondary outcome was to determine when neurocognitive function begins to show distinct characteristics influenced by socioeconomic background and whether these differences evolve over time. To investigate this, neurocognitive assessments were conducted from the 2nd to the 7th check-ups, during which the K-DST and K-ASQ were used interchangeably. Using a three-tiered system, the K-ASQ categorizes outcomes as appropriate or requiring follow-up or further evaluation [19]. 

### Covariates

We aimed to incorporate a thorough range of subject-related information that could potentially influence developmental outcomes. Variables such as HC and feeding type in 4–6 months old, which were anticipated to exhibit a significant correlation with neurodevelopment, were included in our analysis because of their perceived importance.

Anthropometric indices, including weight, height, body mass index (BMI), and HC, were acquired through physical measurements [20]. We utilized data on BW and HC values, along with weight, height, and BMI values at the 7th check-up. Measurement precision was ensured by turning the head horizontally around the upper part of the left ear and the protruding section of the forehead while gently pressing the hair. Standardized scores (z-scores) for height, weight, BMI, and HC in male and female children of varying ages were calculated using lambda for skew, mu for median, and sigma for the generalized coefficient of variation method. For participants aged ≥ 2 years, the 2017 Korean National Growth Charts were employed [21, 22], while the World Health Organization growth standards were utilized for infants and young children aged < 2 years.

Regarding residential status classification, children residing in metropolitan areas (Busan, Daegu, Incheon, Gwangju, Daejeon, and Ulsan) were designated as “metropolitan,” while the remaining regions were categorized as either “city” or “rural,” following the administrative divisions of the Republic of Korea.

Within the scope of the variables used in our analysis, sex, BW, HC, birth year, residence, and breastfeeding status were categorized as nominal variables, whereas age, weight, height, and developmental delay were considered continuous variables.

### Statistical analysis

Quantitative results are expressed as absolute numbers with frequencies and means with standard deviations. A limited number of cases had some missing data; this was < 5% and not enough to interfere with statistical significance. We employed a binary logistic regression model with adjusted odds ratios (aORs) to explore the association between SES and neurodevelopmental function. SES, stratified into low, intermediate, and high tiers, functioned as the independent variable, while developmental delays including various domains, such as gross motor skills, fine motor skills, cognition, language, sociality, and self-care, were the dependent variables. Covariate adjustments included sex, BW, HC z-score, income, birth year, and breastfeeding status. Model 2 introduced the BMI value at the 7th check-up to further refine our understanding of the SES–child development relationship.

As a secondary outcome, we employed generalized estimating equations (GEEs) to examine the evolution of developmental delays in relation to SES across multiple screening timepoints. The GEE, which was applied to analyze longitudinal and correlated response data, proved particularly pertinent for binary responses. SES served as the independent variable, whereas developmental delay (categorized as a recommendation for follow-up and further evaluation) was the dependent variable. Time, a continuous variable, spanned from the 2nd to the 7th check-up. Our analysis, which adjusted for covariates such as sex, birth residence, and birth year, enhanced the precision of our examination of the dynamic relationship between SES and developmental delay through multiple screenings.

We performed a subgroup analysis using logistic regression to elucidate the complex relationship between the independent variables and their influence on the probability of event occurrence. This analysis included crucial factors, such as sex, BW (above or below median average), HC z-score (above or below − 1.65), birth residence (Seoul or metropolitan, city, or rural), birth year (2008–2010 and 2011–2012), and breastfeeding at 4–6 months (breastfeeding, formula feeding, or mixed).

All statistical analyses were performed using SAS 9.4 (SAS Institute, Cary, NC, USA).

## Results

### Characteristics of participants

Table 1 presents the demographic characteristics of the participants. A total of 276,167 participants were categorized into low- (66,325), intermediate- (148,980), and high-SES (60,682) groups. We discovered that 51% of children born each year were female. Examination of variables such as age at the 7th check-up, BW, and HC z-score at the first check-up revealed no significant differences among the SES groups. However, significant differences were observed between the SES groups in terms of birth residence, birth year, and obesity at the 7th examination.

**Table 1:** Basic Sociodemographic characteristics of the participants

Characteristic		Low SES, *n* (%) (*n* = 66,325)	Intermediate SES, n (%) (*n* = 148,980)	High SES, n (%) (*n* = 60,862)
Sex				
	Male	32,603 (49)	73,139 (49)	30,075 (49)
	Female	33,722 (51)	75,841 (51)	30,787 (51)
Age at 7th K-DST assessment^a^		5.78 ± 0.19	5.79 ± 0.18	5.79 ± 0.18
Birth Weight		3.22 ± 0.33	3.22 ± 0.33	3.22 ± 0.33
HC z score at 4–6 mo.^b^		-0.04 ± 0.99	-0.00 ± 0.98	0.05 ± 0.97
	≦ -1.65	33,722 (51)	75,841 (51)	30,787 (51)
	> -1.65	3,474 (5)	6,901 (5)	2,451 (4)
Birth Residence				
	Seoul	10,946 (17)	27,709 (19)	14,510 (24)
	Metropolitan/City^c^	48,294 (73)	111,013 (75)	42,782 (70)
	Rural ^c^	5,843 (9)	9,512 (6)	3,387 (6)
	Missing/Etc. ^d^	1,242 (2)	746 (1)	183 (0)
Birth Year				
	2009	20,307 (31)	37,837 (25)	11,599 (19)
	2010	24,288 (37)	54,712 (37)	20,678 (34)
	2011	21,730 (33)	56,431 (38)	28,585 (47)
Obesity at 66–72 mo.^e^				
	Absence	59,842 (90)	136,700 (92)	56,445 (93)
	Presence	6,483 (10)	12,280 (8)	4,417 (7)
BMI z score at 66–72 mo.^e^		0.098 ± 1.166	0.024 ± 1.120	-0.027 ± 1.092

^a^ Age at 7th K-DST assessment is defined as the age of the participant at the time of the 7th check-up (66-72months)

^b^ Obtained from the first National Health Screening Program for Infants and Children at 4–6 months after birth

^c^ Metropolitan areas are defined as six metropolitan cities (Busan, Incheon, Gwangju, Daejeon, Daegu, and Ulsan), cities as urban areas, and rural areas as non-city areas

^d^ Missing data occurred in 0.79% (2171/276167) of all birth residence data

^e^ Calculated by height and weight obtained from the first National Health Screening Program for Infants and Children at 66–72 months after birth

Abbreviations: BMI: body mass index; HC: head circumference; K-DST: Korean Developmental Screening Test; SES: socioeconomic status

### Main outcome

In children who underwent the 7th K-DST, significant differences in overall neurodevelopmental delays were observed between the high- and low-SES groups. Furthermore, significant differences were found across all six domains (gross motor, fine motor, cognition, language, sociality, and self-care) between the SES groups. These distinctions persisted consistently between the crude and adjusted models, except the self-care domain, in which changes were noted with the application of the adjusted model. Specifically, the low-SES group exhibited a 32.8% (aOR, 1.328; 95% confidence interval [CI], 1.105–1.597) increased likelihood of delays in general development. The most pronounced discrepancies were evident in the cognition (aOR, 1.474; 95% CI 1.327–1.637) and language (aOR 1.455; 95% CI 1.312–1.613) domains (Table 2). Furthermore, when comparing the high- and intermediate-SES groups, discernible differences were evident. These differences, particularly in the cognition (aOR, 1.036; 95% CI, 0.946–1.130) and language (aOR, 1.114; 95% CI, 1.032–1.203) domians, remained statistically significant after adjusting for relevant factors. These disparities manifested a reduced risk compared with that of the low-SES group (Table 2).

**Table 2:** Associations of Socioeconomic Status and Neurocognitive Development

Characteristic	SES	Cohort	Event (%)	Crude OR (95% CI)	aOR (Model 1) ^a^ (95% CI)	aOR (Model 2) ^b^ (95% CI)
Overall	Low	65,349	976 (1.5)	1.597 (1.439–1.772)	**1.336 (1.111–1.606)**	**1.328 (1.105–1.597)**
	Intermediate	147,514	1,466 (1.0)	1.063 (0.964–1.171)	0.984 (0.859–1.126)	0.983 (0.859–1.125)
	High	60,298	364 (0.9)	ref	ref	ref
Gross motor	Low	64,542	1,783 (2.8)	1.388 (1.288–1.495)	**1.271 (1.116–1.447)**	**1.268 (1.113–1.444)**
	Intermediate	145,779	3,201 (2.2)	1.103 (1.031–1.180)	1.064 (0.968–1.169)	1.063 (0.967–1.169)
	High	59,674	1,188 (2.0)	ref	ref	ref
Fine motor	Low	64,060	2,265 (3.5)	1.506 (1.408–1.612)	**1.323 (1.174–1.489)**	**1.320 (1.173–1.487)**
	Intermediate	145,209	3,771 (2.6)	1.106 (1.040–1.177)	1.064 (0.960–1.141)	1.046 (0.959 − 0.141)
	High	59,466	1,396 (2.3)	ref	ref	ref
Cognition	Low	63,270	3,055 (4.8)	1.730 (1.626–1.837)	**1.477 (1.330–1.640)**	**1.474 (1.327–1.637)**
	Intermediate	144,102	4,878 (3.4)	1.212 (1.145–1.282)	**1.129 (1.045–1.220)**	**1.219 (1.045–1.220)**
	High	59,208	1,654 (2.8)	ref	ref	ref
Language	Low	63,174	3,151 (5.0)	1.747 (1.645–1.856)	**1.457 (1.314–1.616)**	**1.455 (1.312–1.613)**
	Intermediate	143,971	5,009 (3.5)	1.219 (1.153–1.289)	**1.115 (1.033–1.203)**	**1.114 (1.032–1.203)**
	High	59,173	1,689 (2.9)	ref	ref	ref
Sociality	Low	64,320	2,005 (3.1)	1.386 (1.292–1.486)	**1.217 (1.077–1.375)**	**1.218 (1.078–1.376)**
	Intermediate	145,381	3,599 (2.5)	1.101 (1.033–1.173)	1.034 (0.946–1.129)	1.036 (0.946–1.130)
	High	59,523	1,339 (2.2)	ref	ref	ref
Self-care	Low	64,841	1,484 (2.3)	1.198 (1.108–1.295)	**1.129 (0.983–1.296)**	**1.128 (0.982–1.295)**
	Intermediate	146,163	2,817 (1.9)	1.009 (0.941–1.081)	0.981 (0.889–1.084)	0.981 (0.889–1.084)
	High	59,721	1,141 (1.9)	ref	ref	ref

^a^ Model 1 is adjusted for sex, birth weight, head circumference at 4–6 months, residence at birth, year of birth, and breastfeeding status at 4–6 months

^b^ Model 2 is adjusted for Model 1 with the BMI of the 7th iteration

Reference: high SES

SES, socioeconomic status; OR, odds ratio; aOR, adjusted odds ratio; CI, confidence interval

### Secondary outcome

**Fig. 2:**
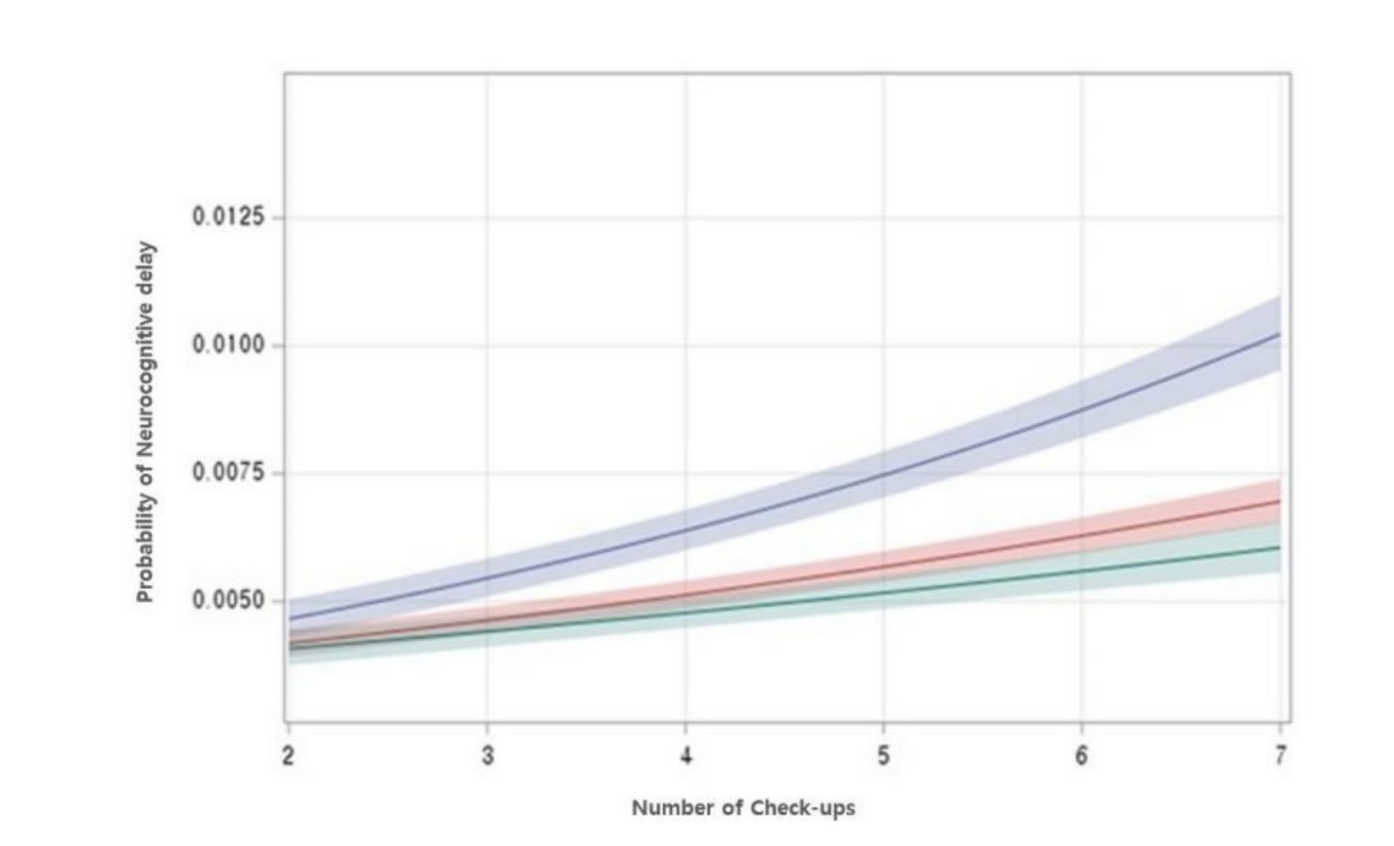
Predicted probability of neurodevelopmental delay according to socioeconomic backgrounds. This figure shows that in the 2nd check-up, there was no difference in neurodevelopmental delay between the low- and high-SES groups; however, a significant difference emerged from the 3rd check-up and continued to grow until the 7th check-up (time estimate, 0.0867; interaction *P* < 0.001). Blue, low SES; Red, intermediate SES; Green, high SES. SES, socioeconomic status

We investigated developmental changes at successive screening intervals, beginning with the second screening, encompassing developmental assessments at 9–12 months. Distinct differences by SES became apparent from the third screening (18–24 months) onward. Both screenings necessitated follow-up assessment. In the presence of an interaction between SES and time to neurodevelopmental delay, the analysis revealed a significant effect of time, with an estimate of 0.0864 (interaction *P* < 0.001). These findings persisted after adjusting for sex, place of birth, and year of birth (estimate 0.0867, interaction *P*-value < 0.001), as illustrated in Fig. 2.

### Subgroup analysis

**Fig. 3:**
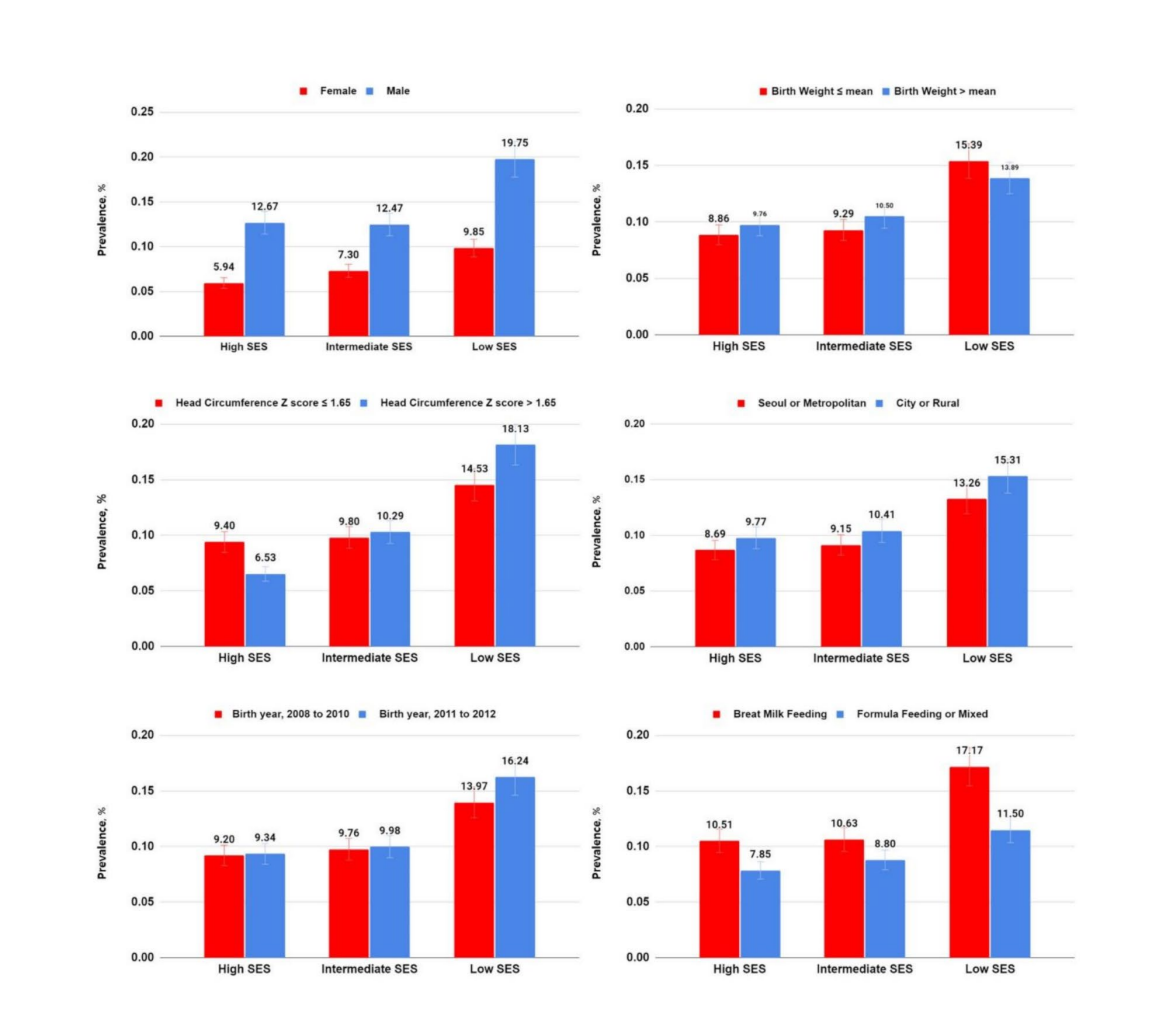
Prevalence of neurodevelopmental delays with socioeconomic status and covariates. Prevalence of neurodevelopmental delays (event/1000 children) according to socioeconomic status groups using covariate variables: (**A**) sex, (**B**) birthweight, (**C**) head circumference at 4–6 months, (**D**) birth residence, (**E**) birth year, and (**F**) breastfeeding status at 4–6 months. SES, socioeconomic status

**Fig. 4:**
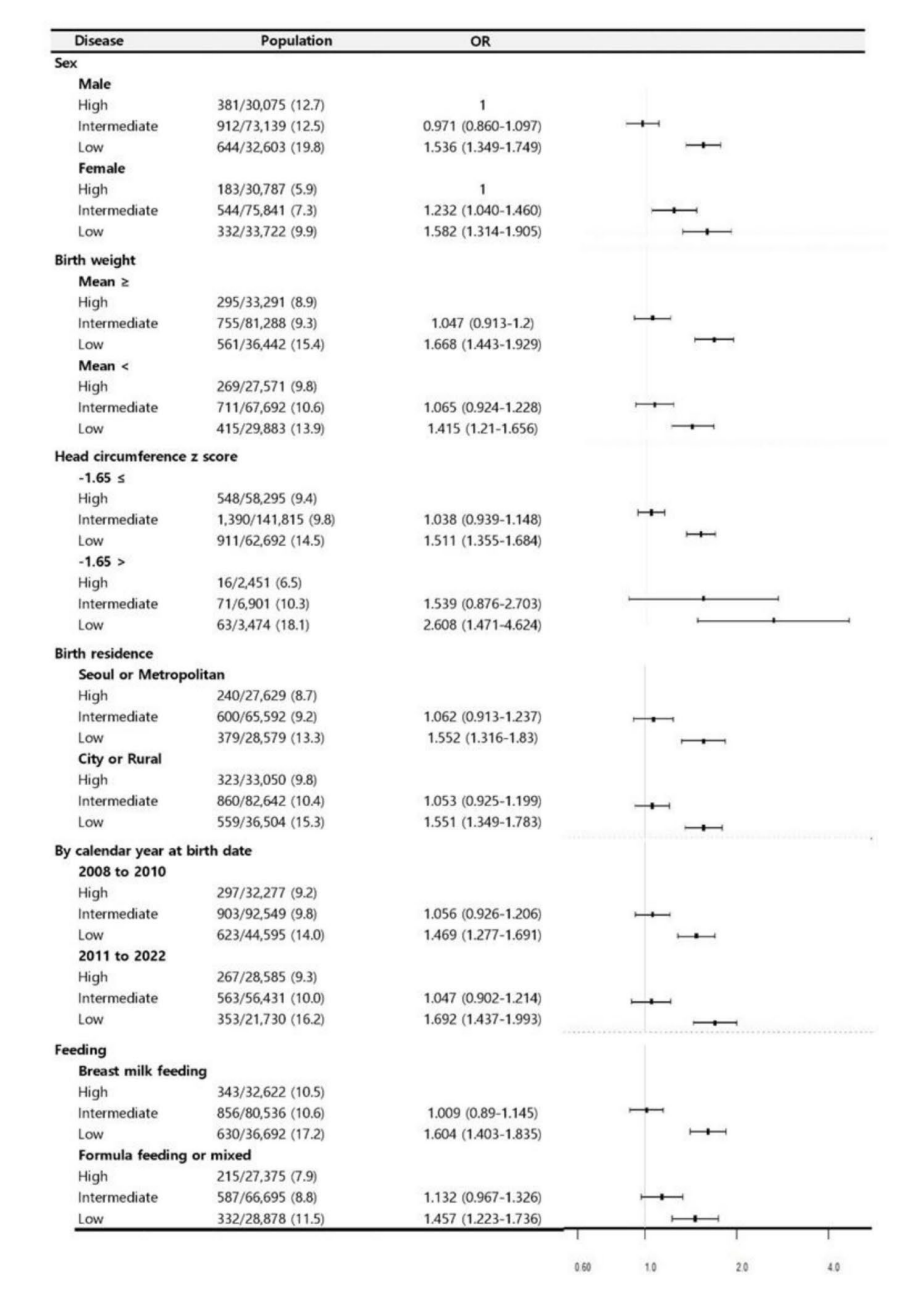
Subgroup analysis of children with neurodevelopmental delays. This figure shows the results of a subgroup analysis of children with neurodevelopmental delays and the association between SES and the prevalence of covariates (sex, birthweight, head circumference, birth residence, year of birth, and breastfeeding status). Reference: high SES. OR, odds ratio; SES, socioeconomic status

In the subgroup analyses, no discernible distinctions were observed between the high- and intermediate-SES groups across the spectrum of measured parameters. Nevertheless, in the low-SES cohort, a substantial disparity was observed in all parameters. There was an increased prevalence of neurodevelopmental delays, regardless of SES, particularly among males, individuals born in cities and rural areas, those did not exclusively breastfed, and those within the temporal window from 2011 to 2012, as shown in Fig. 3. Stratified analyses were conducted to assess the effects of these conditions on neurodevelopmental delays. While neurodevelopmental delays appeared more prevalent in specific groups within the low-SES group than those in the high-SES group, no differences were observed based on sex, BW, HC, or breastfeeding status, except birth year, as depicted in Fig. 4.

## Discussion

This study revealed SES-related disparities in neurodevelopment among preschool-aged children. This nationwide cohort study strongly supported that children born with low SES are at an increased risk of neurodevelopmental delay by age six compared with those born with high SES [23, 24]. 

Cognitive and language skills emerged as salient areas of distinction, although disparities were evident across all developmental domains. Our findings corroborate the well-established notion that SES has a robust impact on various domains of child development. When we examined the impact of these variables on the development of our cohort, we observed similarities with previous research, such as sex, while uncovering divergences exemplified by the role of breastfeeding. 

We also investigated the timeline at which SES-related developmental delays became apparent. Intriguingly, disparities were discernible as early as 9–12 months of age, coinciding with the initiation of developmental screening, and became more obvious by 18–24 months. This temporal analysis provides valuable insights into the trajectory of SES-related developmental differences, thereby enhancing our understanding of the critical periods during which interventions may be most impactful.

The study employed a large, nationally representative cohort, thereby ensuring a substantial and minimally biased sample. A follow-up period of six to seven years permitted a comprehensive assessment of longitudinal developmental changes. To ensure the reliability of our findings, we employed robust statistical adjustments.

The results of studies conducted in the United Kingdom indicate that children from lower socioeconomic backgrounds are at an increased risk of experiencing language delays [5]. Our findings on discrepancies in cognitive and linguistic development during the preschool years are consistent with broader evidence, particularly from cohort and longitudinal studies worldwide, that associate low SES with developmental delays. One crucial mechanism pertains to the early home environment and parent-child interactions. Children from low-socioeconomic status households frequently have fewer opportunities for enriching language exposure and cognitive stimulation, which are essential for early neurodevelopment. A paucity of linguistic input and a diminished quality of caregiver-child interactions have been demonstrated to impede both cognitive and linguistic development [25]. For example, a cohort study conducted in the United Kingdom underscored the pivotal role of the early home environment in shaping language skills by middle childhood. This finding corroborates our own study, in which significant delays in language development among children from low-income backgrounds were observed as early as age two. These delays are likely the result of reduced access to early educational resources and lower levels of cognitive stimulation in the home environment. This underscores the need for early interventions that improve parental support and home learning conditions [26]. Cognitive developmental delays appear to occur in infancy, with discernible differences in vocabulary and picture similarity [27]. These early disparities, particularly in language and cognitive domains, are precursors to broader discrepancies, including lower intelligence quotient (IQ) and divergent academic performance [5, 28].

The cognitive and language development of children from low SES backgrounds is influenced by a number of key factors, including parental education, the home environment, caregiver-child relationships, and language exposure. Chronic stress, which is prevalent in low-income households, can have a detrimental impact on parental mental health and subsequently reduce the quality of these interactions, which in turn contributes to developmental delays. The implementation of parental support programs and mental health services could serve to mitigate the adverse effects of stressors on the home environment, thereby facilitating more optimal developmental outcomes [29]. These previous studies have shown that various socioeconomic factors are related to children's development [30–32]. This study showed that the association between SES and development remained effective even after adjusting for confounding factors such as region of birth, infant developmental status, type of feeding, or certain biological predispositions. The relationship between SES and cognitive development is influenced by geographic boundaries, economic inequality, [33–35] and nutritional status [34, 35].

Neuroimaging studies have demonstrated that children from high and low SES backgrounds exhibit distinct differences in brain activity patterns, providing a neurobiological explanation for the disparities in cognitive and language skills. Children from families with higher SES levels tend to demonstrate increased brain activity in regions associated with mathematical and linguistic processing, whereas children from lower SES backgrounds exhibit heightened activity in areas linked to spatial processing. These neurobiological differences are likely influenced by the combined effects of environmental stress and reduced cognitive stimulation, thereby underscoring the importance of interventions that enhance cognitive and language development in low-income families [36].

In contrast to earlier studies, this research found that breastfeeding did not significantly impact on development. While breastfeeding is acknowledged for its myriad advantages, a nuanced perspective must be entertained, exercising cautious optimism regarding its role in preventing developmental delays. Long-chain polyunsaturated fatty acids in breast milk play a pivotal role in facilitating optimal brain development, as evidenced by consistent research highlighting the advantageous impact of breastfeeding on neurological development and superior academic performance. Empirical evidence supports the increased neural volume and activity in critical brain regions associated with neurological function in breastfed children. Additionally, breastfeeding fosters an enhanced mother–child bond, exerting a beneficial influence on neurodevelopment [17]. Other factors influencing neurodevelopment, such as atopic dermatitis, torticollis, and soy milk feeding, merit consideration in future studies and subgroup analyses [37–39]. Our findings regarding breastfeeding diverge from conventional wisdom. While breastfeeding is acknowledged for its myriad advantages, a nuanced perspective must be entertained, exercising cautious optimism regarding its role in preventing developmental delays. However, this optimism is tempered by the conjecture that breastfeeding efficacy may be influenced by intricate mechanisms related to micronutrient availability and maternal nutritional status.

Developmental screening plays a critical role in identifying developmental delays at the earliest possible juncture and facilitating timely interventions. The present study found an elevated prevalence of developmental delays in lower-SES groups at an earlier age, accentuating the potential societal advantages of directing attention toward developmental delays in these cohorts. While the overarching objective of this screening initiative may not be universally acknowledged, its primary objectives include augmenting the likelihood of detecting developmental delays through standardized instruments, expediting referrals for specialized assessments [40]. This proactive strategy, extending beyond mere developmental surveillance, is of paramount importance for enabling early interventions and mitigating the protracted societal ramifications associated with developmental delays. Global implementation of such screening programs underscores their significance in addressing developmental delays and fostering timely interventions to improve long-term outcomes.

It is important to consider the limitations of this study. Although the study was based on extensive datasets with multiple calibration variables to mitigate selection bias, the inherent lack of numerical precision of the developmental test posed a methodological challenge. Applying developmental tests that show quantitative outcomes, rather than screening tests or binary categorization, would have yielded more meaningful results. Analysis using continuous data as the outcome variable may reveal more detailed relationships between SES and neurodevelopmental scores. Moreover, the absence of developmental timeline data from birth to the final assessment at 66–71 months imposed constraints on the comprehensive tracking of developmental sequences. Next, the utilization of SES values only at birth may not have precisely captured changes over the 6-year study period. Additionally, the transition from the K-ASQ to the K-DST as a developmental screening test may have influenced accuracy. Furthermore, as both instruments depend on parental input, there is a possibility of subjective bias, which could compromise the precision of developmental assessments in comparison to those conducted by professionals. In the absence of K-DST data, the study resorted to the K-ASQ, resulting in numerical inconsistencies despite efforts to align the results closely. The study has potential recall bias for a few surveys and overlooked key educational measures, such as day care or preschool attendance, which play a pivotal role in the comprehensive assessment of child development. The study excluded approximately 70% of children due to strict inclusion criteria, which raises concerns about the representativeness of the population studied. This exclusion may have introduced systematic bias between respondents and non-respondent such as sex ratio discrepancy. Finally, the study did not account for genetic factors, such as parental IQ, which could influence both SES and child neurodevelopment, and the lack of consideration of these factors may limit the scope of the findings.

In conclusion, SES significantly influences developmental delays in preschool domains, in cognitive and language skills, with these effects intensifying over time. This underscores the critical importance of early screening and intervention, particularly for children from low SES backgrounds. The findings advocate for a multifaceted approach, combining direct interventions with broader socioeconomic support, to address these disparities. In addition, the study highlights the need for future research into the mechanisms behind SES's impact on development and the potential benefits of educational interventions.

## Electronic supplementary material

Below is the link to the electronic supplementary material.

Supplementary Material 1

## Data Availability

No datasets were generated or analysed during the current study.

## Abbreviations

BMI: body mass index
HC: head circumference
K-DST: Korean Developmental Screening Test
SES: socioeconomic status
