# Supplementary material for: Early onset and increasing disparities in neurodevelopmental delays from birth to age 6 in children from low socioeconomic backgrounds
Source: J Neurodev Disord. 2024 Nov 5;16:60. doi: 10.1186/s11689-024-09577-2 (PMC11536651; doi:10.1186/s11689-024-09577-2)
Supplement: Supplementary file 1 — Supplementary Material 1 [file 11689_2024_9577_MOESM1_ESM.docx]

**Online-only Material**

**eFigure 1. National Health Screening Program for Infants and Children**

**eFigure 1. National Health Screening Program for Infants and Children**


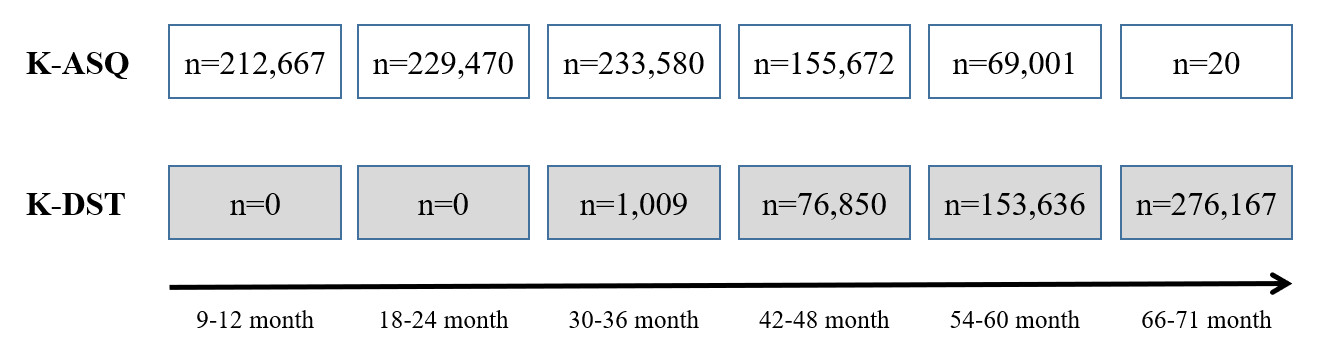


This figure shows the timing of developmental screening tests under the National Health Screening Program for Infants and Children. The K-ASQ, which was used until 2013, was used for children aged 4-60 months, and the K-DST, which has been used since 2014, was used for children aged 9-71 months.

K-ASQ, Korean Infant and Toddler Ages and Stages Questionnaire; K-DST, Korean Developmental Screening Test
